# Non-Endoscopic Screening for Barrett’s Esophagus Using a DNA Methylation-Based Assay: Real-World Experience in 11,991 Patients

**DOI:** 10.3390/diagnostics16213566

**Published:** 2026-10-29

**Authors:** Michael S. Smith, William T. Chen, Paul S. Panzarella, Ivy T. Le, Suman Verma, Victoria T. Lee, Lishan Aklog

**Affiliations:** 1 Division of Gastroenterology, Department of Medicine, Icahn School of Medicine at Mount Sinai, New York, NY 10029, USA; michael.smith1@mountsinai.org; 2 Warren Alpert Medical School, Brown University, Providence, RI 02912, USA; william.chen@gialliance.com; 3 University Gastroenterology, 33 Staniford, St Providence, RI 02905, USA; 4 Center for Advanced GI, Maitland, FL 32751, USA; ppanzarella@gmail.com; 5 Lucid Diagnostics Inc., New York, NY 10017, USA; ile@luciddx.com (I.T.L.); vtl@pavmed.com (V.T.L.); la@luciddx.com (L.A.)

**Keywords:** Barrett’s Esophagus, esophageal adenocarcinoma, gastroesophageal reflux disease, non-endoscopic, screening, methylation

## Abstract

***Background****:* Barrett’s esophagus (BE), defined by specialized intestinal metaplasia (SIM), is the recognized precursor to esophageal adenocarcinoma (EAC). Despite established screening guidelines for high-risk populations, uptake of endoscopic screening remains low. We report the results of early commercial use of a non-endoscopic BE screening strategy, EsoGuard^®^ (EG), a molecular biomarker assay performed on esophageal cell samples collected via a companion swallowable balloon capsule device (EsoCheck^®^, EC). ***Methods****:* We retrospectively analyzed the feasibility and tolerability of EG/EC as a non-endoscopic approach to BE evaluation in patients tested from January 2023 through June 2024. Multivariable logistic regression was used to evaluate risk factors associated with positive EG results. ***Results****:* Among 11,991 patients tested, 11,355 (94.7%) had successful cell collection with the EC device, with procedures averaging less than 2 minutes and no device-related adverse events reported. Over 95% of surveyed patients would recommend the test to others. Overall, 93.1% (10,566/11,355) of collected samples had binary EG results, among which 17.8% had positive assay results suggesting the presence of BE. Positivity rates increased with age, with age > 50 years as the strongest individual predictor of a positive EG result. ***Conclusions***: These data represent the largest real-world experience with EG and EC to date, demonstrating high tolerability and no reported device-related adverse events. The findings support the feasibility of large-scale implementation and suggest that EG/EC use may improve access to guideline-recommended BE screening across diverse healthcare settings.

## 1. Introduction

The incidence of esophageal adenocarcinoma (EAC), the most common esophageal cancer in the U.S., has increased greatly over recent decades, with 5-year survival remaining poor at about 20% [1,2]. Barrett’s esophagus (BE), a condition of specialized intestinal metaplasia (SIM) within the tubular esophagus, is the direct precursor to EAC [3,4]. In contrast to the lethality of EAC, BE is treatable endoscopically. Current U.S. gastroenterology guidelines recommend screening in high-risk patients with multiple well-defined risk factors. These include symptoms of gastroesophageal reflux disease (GERD), male sex, white race, age > 50 years, obesity, tobacco smoking, and family history of BE or EAC [5,6].

Unfortunately, in current practice, only approximately 10% of eligible patients undergo endoscopic evaluation for BE, an issue that is likely multifactorial in nature [7,8]. Patient failure to recognize risk factors including GERD, limited knowledge of screening recommendations by primary care physicians, and patient concerns regarding the logistics and risks of upper endoscopy (esophagogastroduodenoscopy, EGD) contribute to the low rates of endoscopic screening and sub-optimal disease detection [9,10,11]. BE is estimated to be present in approximately 10% of U.S patients with GERD, likely rising with additional risk factors; however, because few screen-eligible patients undergo EGD, most cases remain undiagnosed [8,12]. Significant advances have been made over the last decade in the development of non-endoscopic biomarker-based tests that can serve as adjunctive tools for the diagnosis of BE. The 2022 guideline updates from the American College of Gastroenterology (ACG) and clinical practice updates from the American Gastroenterological Association (AGA) describe “minimally invasive non-endoscopic swallowable cell collection device(s) combined with biomarkers” as an alternative to endoscopy for BE screening in high-risk patients [5,6]. Within this paradigm, non-endoscopic cell collection and biomarker-based testing serves as an initial in-office triage step, directing patients to either (a) medical management if test results are negative or (b) EGD with or without biopsies if positive, for confirmation and histopathologic staging.

The first commercially available molecular biomarker test in the U.S. to support the diagnosis of BE is the EsoGuard^®^ (EG) Esophageal DNA Test (Lucid Diagnostics Inc., New York, NY, USA). EG uses targeted next-generation sequencing (NGS) to detect patients with cellular changes associated with BE. The assay is performed on distal esophageal cell samples collected non-endoscopically using EsoCheck^®^ (EC), an FDA 510(k)-cleared swallowable tethered balloon-capsule device (K230339; Lucid Diagnostics Inc., New York, NY, USA), which can be administered in-office without sedation. This assessment strategy is intended to increase BE screening uptake and disease detection in high-risk populations without disproportionate use of endoscopic resources. We report 18 months of real-world feasibility and tolerability data from nearly 12,000 consecutive patients who underwent EG/EC as a first stage of clinically indicated BE screening.

## 2. Materials and Methods

### 2.1. Data Collection and Participants

A retrospective cross-sectional analysis was conducted including patients who underwent EC esophageal cell collection and EG biomarker testing to screen for BE between January 2023 and June 2024. This study protocol was approved by the Western Institutional Review Board (approval Code: 20244309, approval Date: 25 October 2024), with the Board finding that the study meets requirements for waiver of consent under 21 CFR 50.22. Consecutive patients who underwent EC administered by Lucid Diagnostics personnel were included, with baseline characteristics and procedure data recorded in the central laboratory database as part of standard practice. Standard operating procedures for EC device administration training were followed, including a minimum of 10 proctored cell collections to demonstrate basic competency before personnel were allowed to perform independent (non-proctored) cell collections. Data for individuals who underwent testing performed by their own physicians or affiliated staff were not readily available in a deidentified manner and therefore were not included in this analysis.

Patient demographics, BE risk factors, details of the EC cell collection procedure (namely procedure duration, patient gag response, successful vs. unsuccessful collection, and any adverse events), and EG results were acquired from the central laboratory database in an aggregate fashion without specific patient identifying information. Although the presence or absence of GERD symptoms was captured in the database, the duration of symptoms was not. Results from a voluntary patient survey on the cell collection experience were also obtained from the central laboratory database. The patient’s gag response (GR) during cell collection was assessed by the EC device administrator on a five-point scale (1 = minimal to no gag, 5 = unable to complete the cell collection) and detailed in Appendix A Table A1.

As an FDA 510(k)-cleared device, issues for EC pertaining to patient safety, device malfunctions/failures, and usability have been tracked in compliance with Medical Device Reporting (MDR) requirements. Event documentation was performed in accordance with Lucid Diagnostics’ manufacturing and quality management systems (QMS) requirements. Data pertaining to EC safety during the study timeframe also was collected from the QMS system. Adverse events and device malfunctions were identified through documentation by the device administrator at the time of cell collection and through complaints and reports received by the manufacturer. Patients were not systematically contacted after the procedure for safety follow-up. Malfunctions were further classified according to whether they were identified before patient exposure (i.e., during pre-administration function checks) or during or after device administration.

### 2.2. EsoCheck (EC) Esophageal Cell Sample Collection

**Figure 1:**
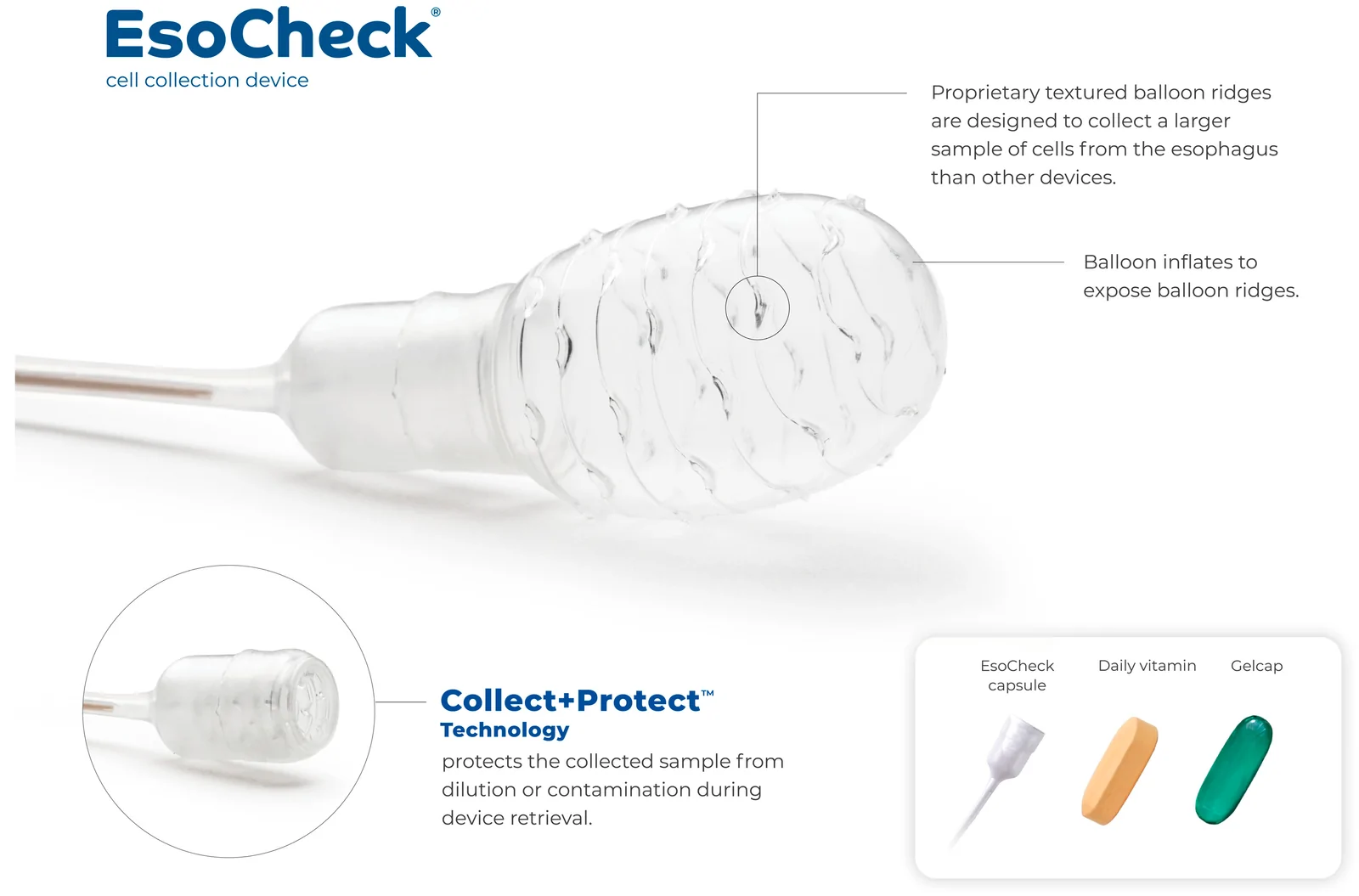
The EsoCheck^®^ balloon capsule device including Collect + Protect™ technology.

The EC device is indicated for use in non-endoscopic collection and retrieval of surface cells of the esophagus in the general population of adults and adolescents 12 years of age and older [13]. The balloon capsule features Collect + Protect technology™ for targeted sampling (Figure 1). The device is swallowed in encapsulated form, after which the balloon is inflated by passing air through the hollow tether using a syringe. Gentle pressure on the tether generates cell sample collection along the textured surface of the inflated balloon, as it is withdrawn through the distal 5 cm of the esophagus. The balloon is then syringe-deflated into the capsule prior to device retrieval, protecting the cell sample from dislodgement and contamination in the upper esophagus and oropharynx. This EC cell collection process follows the device instructions for use and has been described in previous publications [14,15,16].

**Figure A1:**
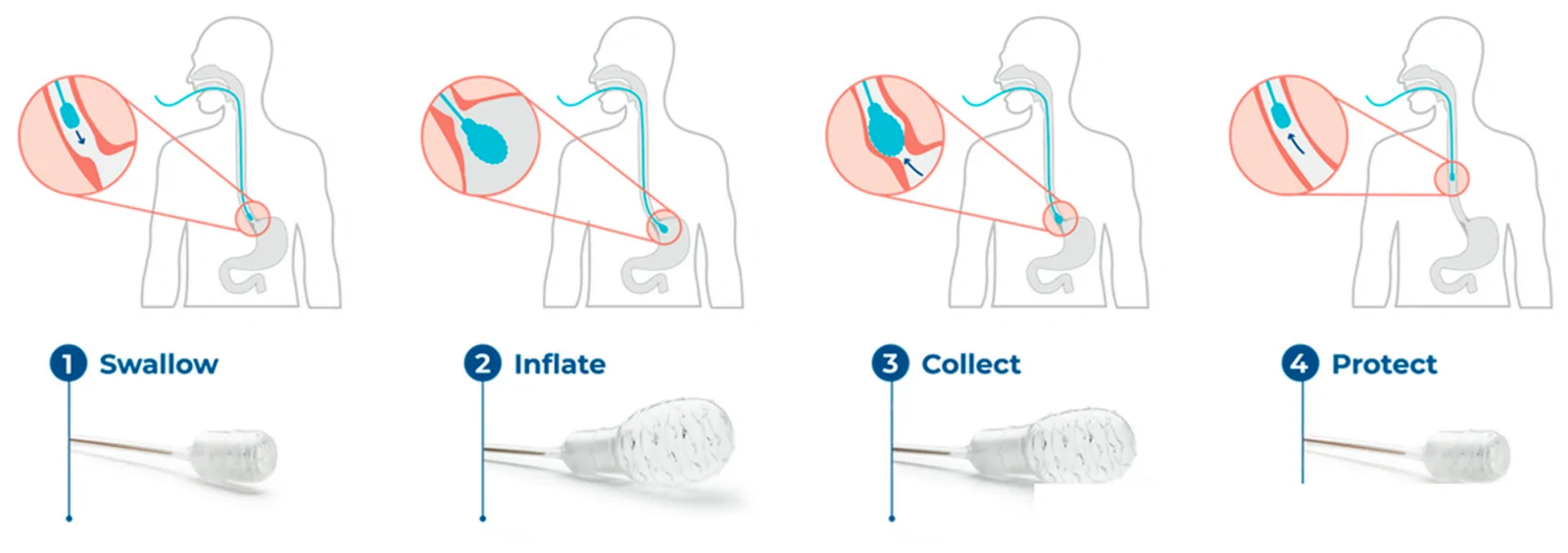
EsoCheck cell collection procedure.

In brief, the patient swallows the capsule, which esophageal peristalsis carries into the stomach. Distance markings on the catheter help the administrator determine when the capsule has likely reached the stomach, at which point the balloon is inflated with air via a syringe at the proximal end of the catheter. The device is then gently withdrawn until resistance indicates contact with the EGJ. The balloon is slightly deflated to allow atraumatic passage across the EGJ, and textured ridges on its surface circumferentially collect cells as it is withdrawn across 5 cm of the distal esophagus, measured using the distance markers. The balloon is then inverted back into the capsule using the syringe, protecting the specimen from dilution and oropharyngeal contamination during removal (Appendix A Figure A1).

Administration of EC can be performed by any trained personnel, including physicians and non-physician healthcare providers such as nurse practitioners, registered nurses, and medical assistants. Collection can occur in any office setting, including a clinic, at health fairs, or at designated commercial test centers.

### 2.3. EsoGuard Assay

EG is a laboratory-developed test that detects DNA methylation at 31 CpG sites on the Vimentin (*VIM*) and Cyclin A1 (*CCNA1*) genes. EG has been clinically validated to detect disease along the full BE progression spectrum, up to and including EAC, in four independent studies (two multi-center case–control and two prospective cohort studies) [14,15,17,18].

All EG testing was performed at LucidDx Labs (Lake Forest, CA, USA), the clinical laboratory of Lucid Diagnostics, which is accredited by the College of American Pathologists (CAP), certified under the Clinical Laboratory Improvement Amendments (CLIA), and approved by the New York State Clinical Laboratory Evaluation Program (NY-CLEP). Following cell collection, EC samples underwent DNA extraction, bisulfite conversion, and polymerase chain reaction amplification in triplicate of targeted VIM and CCNA1 regions, followed by NGS and bioinformatic quantification of methylation across the 31 CpG sites. Each replicate is called positive if either methylated *VIM* (m*VIM*) or methylated *CCNA1* (m*CCNA1*) meets the validated cutoff, and the final EG result is assigned by majority rule (at least two of three replicates). This binary framework enhances interpretability and clinical actionability. The assay has been validated analytically in accordance with CAP, CLIA, and NY-CLEP requirements, as previously described [19].

A positive result indicates aberrant DNA methylation consistent with changes along the BE spectrum (metaplasia, dysplasia, or neoplasia) and should be followed by EGD for visual examination and staging, whereas a negative result indicates a low likelihood of BE or EAC. Samples with insufficient DNA or failed quality checks are reported as DNA Quantity Not Sufficient (QNS) or Unevaluable, respectively.

### 2.4. Outcome Measures

Outcome measures for EC include device tolerability, acceptability, and safety. Device tolerability was assessed using two metrics: (1) the proportion of successful cell collections compared to the total attempted, and (2) the patient’s gag response score during sample collection. EC acceptability was assessed by optional, anonymous patient surveys issued after completion of the cell collection procedure. The surveys collected subjective measures of pre-procedure anxiety, ease of the cell collection, level of discomfort, whether the patient would recommend the test to others, and whether the patient would be willing to repeat the test if clinically indicated. Responses were recorded on a 10-point scale with 1 being the best and 10 the worst score.

EC safety was assessed through reported device malfunctions/failures and adverse events. Events were categorized according to class of malfunction or failure, and any consequences to patient health and welfare, if applicable. EG results were evaluated to assess potential population trends; the effects of individual risk factors as predictors for EG positivity were explored using predictive models.

### 2.5. Statistical Analysis

Summary statistics were performed for patient baseline characteristics and EC procedure characteristics. Means and standard deviations are presented where applicable. The Chi-square test was used for assessing differences between categorical variables.

Multivariable logistic regression models were constructed to develop a predictive algorithm for estimating the probability of a positive EG result. The model included the most relevant available BE/EAC risk factors: presence of symptoms associated with GERD, age > 50, male sex, white race, smoking history, obesity (body mass index (BMI) ≥ 30), and family history of BE/EAC. Internal validation of the model’s discriminative ability (c-statistic) was performed using Harrell’s optimism-corrected bootstrap method [20]. The model was first fit to the full dataset to obtain the apparent c-statistic. It was then refit in each of 10,000 bootstrap samples drawn with replacement, and each refit model was evaluated in both its bootstrap sample and the original dataset. The mean difference between these values (the optimism) was subtracted from the apparent c-statistic to obtain the optimism-corrected estimate. Bootstrap resampling was used only to estimate optimism in model discrimination and was not used to estimate the regression coefficients or their confidence intervals. Odds ratios (OR) are reported to two decimal places and area under the curve (AUC) is reported to three decimal places; all other numbers are reported to a single decimal place.

## 3. Results

### 3.1. EC Performance and Acceptability

**Figure 2:**
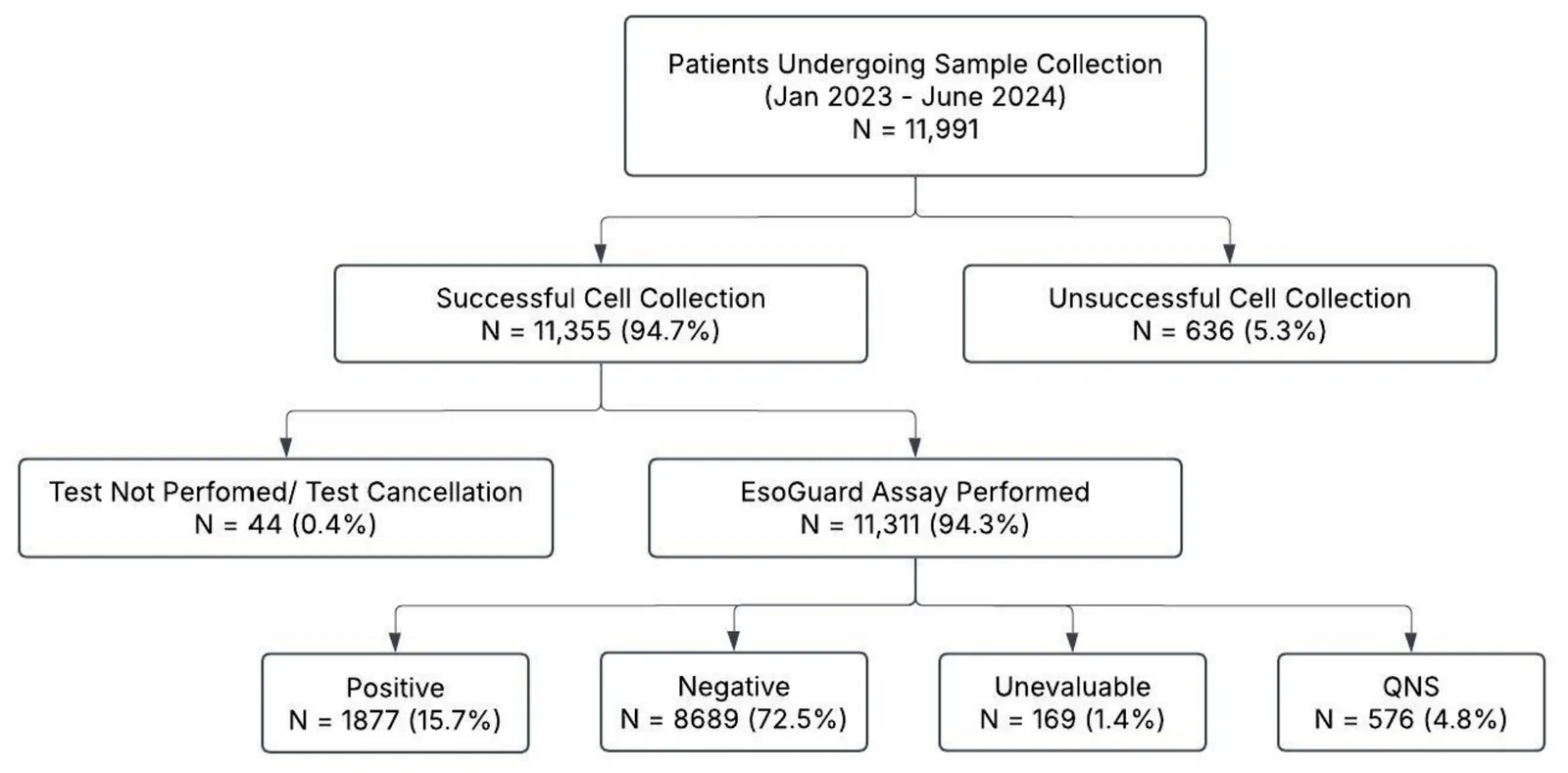
Flow diagram of patients undergoing EC cell sampling by Lucid personnel from January 2023 to June 2024. QNS = DNA Quantity Not Sufficient.

Between January 2023 and June 2024, a total of 11,991 patients underwent EC cell collection by Lucid Diagnostics personnel (Figure 2). Among these, 94.7% (11,355/11,991) successfully produced a cell sample for analysis. Cell collection failed due to gagging in 5.3% of patients (n = 636). During the study period, only 12 patients with an unsuccessful initial cell collection (12/636; 1.9%) underwent repeat testing, which yielded a negative EsoGuard result in five, a positive EsoGuard result in three, and four unsuccessful repeats. EC success rates were highly consistent over time, with quarterly successful cell collection rates ranging from 93.5% to 97.1%. Technical success rates did not vary significantly from month to month, or by individual sample collectors. Newly hired staff were noted to reach technical success rates matching their steady state almost immediately after completion of training.

Patient demographics and BE risk factors were analyzed for patients who successfully provided cell samples to the laboratory (N = 11,355; Table 1). The most prevalent risk factors were white race (72.9%) and male sex (68.8%).

Among patients who provided a cell sample, most (89.2%; 10,130/11,355) experienced no more than minimal to moderate gagging during cell collection (GR score ≤3). The average cell collection time was 1.9 ± 0.9 minutes, and 98% (11,131/11,355) of cell collections were completed in less than 5 minutes. Satisfaction surveys were obtained from 586 patients (Appendix A Table A2). While most patients expressed at least some degree of pre-procedure anxiety, 82.4% of survey respondents reported low levels of discomfort (score ≤ 3) and 82.3% reported minimal difficulty swallowing the device (difficulty score ≤ 3). Additionally, 95.6% of respondents stated they would recommend EC to others in need of BE screening, and 94.6% would be willing to repeat the test if clinically indicated.

### 3.2. EC Device Complaints/Malfunctions and Safety

Twenty-nine device malfunctions were reported in the QMS during the study period (0.24% of 11,991 attempted collections; Table 2). Five (0.04%) were identified before patient exposure during routine pre-administration function checks, and the device was replaced before use. The remaining 24 (0.20%) occurred during or after administration: 22/29; 75.9% involved incomplete balloon encapsulation on removal, and 2 involved balloon microtears. Incomplete encapsulation was managed with repeat cell collection using a new device. No patient harm was reported in association with any malfunction. No major device failures have been reported to date, including no instances of capsule detachment from the silicone tether.

### 3.3. EG Results

Among 11,355 cell samples collected, binary test results were issued for 10,566 (93.1%; Figure 2). Reasons for laboratory non-results included: (a) 44 samples (<1%) that could not be analyzed either due to sample integrity issues or test cancellation by the provider; (b) 576 samples (5.1%) that had insufficient DNA for analysis (reported as QNS); and (c) 169 samples (1.5%) that failed quality check (reported as Unevaluable). The EG positivity rate for all patients who attempted testing was 15.7% (1877/11,991); the positivity rate among laboratory samples with a first-pass yield was 17.8% (1877/10,566). Two patients with an initial test cancellation were retested; one result was negative and the other was QNS. There were 97 patients with an initial QNS or unevaluable result who underwent repeat testing, 77 (79.4%) of whom obtained a binary EsoGuard result on the second attempt. Among these, 63/77 (81.8%) were EsoGuard-negative and 14/77 (18.2%) tested positive. Seven patients with an initial binary result underwent physician-ordered repeat testing during the 18-month study period, all of whom again obtained binary results.

### 3.4. Predictors of EG Positivity

**Figure 3:**
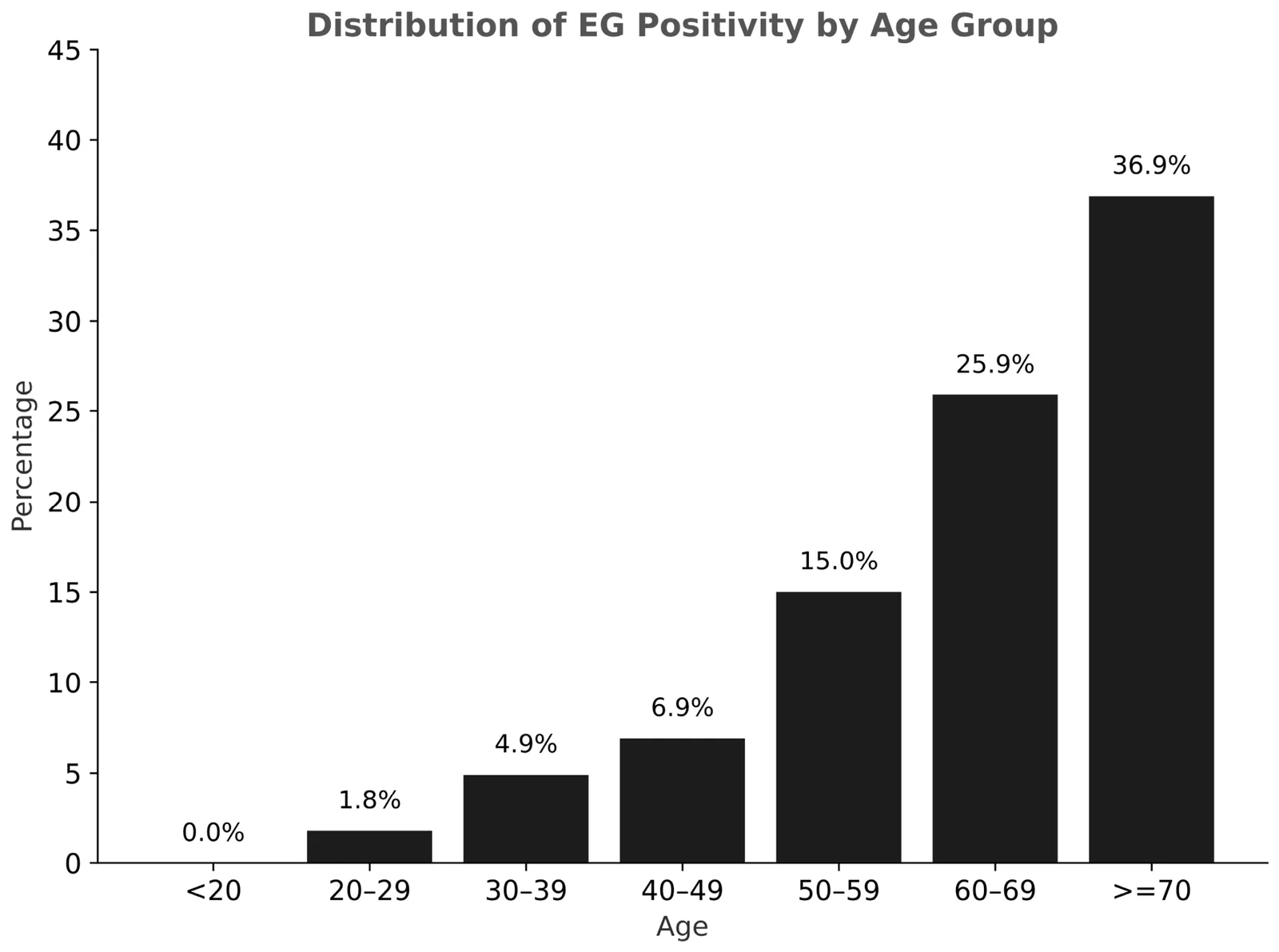
Rate of EsoGuard positivity across different patient age groups (in years) among the commercially tested population.

A multivariable logistic regression analysis was performed to evaluate individual BE risk factors as predictors of EG positivity (Table 3). Age > 50 years was shown to be the most predictive risk factor, with an OR of 5.59 (95% CI: 4.86–6.44, *p*-value <0.0001). EG positivity rates increased with age across the full analysis population (Figure 3). The presence of a family history of BE/EAC (OR: 1.42, 95% CI: 1.14–1.78, *p*-value = 0.0020) and tobacco smoking history (OR: 1.33, 95% CI: 1.20–1.48, *p*-value = <0.0001) were also associated with EG positivity, whereas obesity (BMI ≥ 30) was inversely associated with EG positivity (OR: 0.82, 95% CI: 0.74–0.90, *p*-value = 0.0001).

## 4. Discussion

This real-world data analysis represents the largest feasibility and tolerability study of non-endoscopic BE screening with EG/EC, encompassing nearly 12,000 patients. These data support rapid sample collection and high tolerability of the EsoCheck device, with a low rate of reported device-related events. Binary EG results were issued for 10,566 of the 11,355 successfully collected samples (93.1%), representing 88.1% of the 11,991 patients in whom collection was attempted. This finding suggests that EG/EC provides a practical and feasible non-endoscopic in-office approach to EGD triage for BE diagnosis. Patients who underwent repeat testing (n = 118; 1.0%) were retained in the analysis to avoid selection bias, as repeat testing was not random and excluding these patients could have altered the risk profile of the study population. For the seven patients with two binary results, each result was retained as a separate test event, as the two results came from separate cell collections at different time points. Because these repeat observations represent less than 0.1% of the observations in the regression model, they would not be expected to materially affect the results.

EC’s balloon-capsule design with a durable silicone tether distinguishes it from other non-endoscopic cell collection devices such as Cytosponge™ (Medtronic, Galway, Ireland), EsophaCap^®^ (Lucid Diagnostics Inc., New York, NY, USA), and EndoSign^®^ (Cyted Ltd., Cambridge, UK), which use sponge-on-a-string (SoS)-based technology. These SoS devices consist of mesh spheres compressed within gelatin capsules that expand upon deployment and collect surface mucosal cells from the gastric cardia, entire length of the esophagus, and the oropharynx. SoS devices typically require approximately 10 min for sample collection, as the gelatin capsule must dissolve in the stomach before retrieval [21,22,23]. Sponge detachment during retrieval, necessitating endoscopic removal, has led to FDA recalls of both the Cytosponge™ and EsophaCap^®^ devices [21,22,24,25]. In the present real-world dataset, EC sample collection was completed in under five minutes in 98% of cases, with an overall administration success rate of 95%, which is within the range reported for other non-endoscopic collection devices [22,23,24,26]. This study was not designed as a head-to-head comparison, and published studies of SoS devices differ from the present analysis in study design, procedural protocols, and operator training. Prospective comparative studies would be needed to characterize any meaningful differences in procedural performance, safety, or tolerability between devices. Nonetheless, the rapid cell collection times observed for EsoCheck support the feasibility of EsoGuard testing in high-throughput settings, such as health fairs, and allow for easier integration into busy outpatient practice workflows. Importantly, no device-related adverse events have been reported and malfunctions occurring during or after administration were infrequent (0.2%) and were not associated with reported patient harm, supporting a highly favorable safety profile for EsoCheck in routine clinical use.

The analytical and clinical performance characteristics of the EG/EC test (including sensitivity, specificity, positive predictive value [PPV], and negative predictive value [NPV]) have been rigorously established and previously were published in independent clinical validation studies. This study is intended to complement those prior efforts by demonstrating the feasibility, adoption, and performance of the testing paradigm in real-world clinical workflows. It is notable that in this real-world experience, 17.8% of laboratory samples with a first-pass yield were EG-positive, aligning with a previously published prospective single-arm screening study that reported a 19% EG positivity rate and 8% BE prevalence on confirmatory EGD [15].

Age > 50 was a strong predictor of EG positivity (OR: 5.59) in multivariable logistic regression models. This aligns with the published literature demonstrating higher BE prevalence in older age groups (most notably among those aged > 60 years) [27]. Tobacco smoking and family history of BE or EAC also were predictors of EG positivity, with respective odds ratios of 1.33 and 1.42. However, these values were lower than expected, particularly for patients with a positive family history, as prior studies report odds ratios up to 12.2 in that cohort, and 1.8 for SIM among smokers [28,29]. This discrepancy may stem from inaccurate risk factor reporting, as patients often lack familiarity with full family medical history, and the literature suggests nearly 13% of smokers and 6% of former smokers have withheld their smoking status from their healthcare providers, potentially due to perceived stigma or insurance reasons [30]. Obesity (BMI ≥ 30) was also unexpectedly inversely associated with EG positivity (OR: 0.82; 95% CI: 0.74–0.90), despite being an established risk factor for BE and EAC. However, among patients with GERD, BMI has shown little independent predictive value for BE, and central adiposity, rather than overall BMI, appears to drive this risk [31]. BMIs calculated from inaccurate or outdated patient-reported height and weight may have also contributed to this finding.

The finding that the presence of GERD symptoms was not a statistically significant predictor of EG positivity was unexpected, given that reflux is the presumed underlying mechanism driving BE metaplasia. However, a growing body of literature suggests that symptoms may be a poor indicator for the presence of chronic GERD. Studies have shown that a substantial proportion of asymptomatic individuals may have esophagitis, and up to 50% of patients who ultimately develop esophageal adenocarcinoma do not report frequent GERD symptoms beforehand [32,33]. Reliance on patient self-reporting of GERD symptoms also may have contributed to the lack of apparent association with EG positivity. While heartburn is commonly identified as a manifestation of GERD, atypical or extra-esophageal symptoms may be underrecognized or dismissed. Conversely, intermittent or isolated episodes of heartburn may be overreported as GERD in the absence of chronic or clinically meaningful disease. Either scenario could have attenuated any true association between GERD symptoms and EG positivity.

Key study limitations include both the retrospective, observational study design and the reliance on self-reported risk factors, which may introduce misclassification and social desirability bias (e.g., GERD, smoking, BMI) despite the large sample size. Limiting the cohort to patients tested by Lucid Diagnostics personnel also may have introduced selection bias. However, as more than 80% of total commercial tests performed during the study period were conducted by the same employed personnel, the findings remain broadly representative of the commercial testing population. Of the 1425 (11.9%) whose initial attempt did not yield a cell sample or a binary EsoGuard result, we reported outcomes for the 111 who underwent a second attempt. However, because these data were obtained from laboratory records rather than patient medical records, we could not determine how many patients proceeded directly to EGD or deferred further testing after the initial failure. This has potential implications for the patient journey and provider workflow. Notably, EsoGuard is indicated for patients who would otherwise be unlikely to undergo endoscopic screening through traditional care pathways. Patients who did not complete testing therefore represent a missed screening opportunity rather than a loss relative to standard care, in which most screen-eligible patients are never screened. Additionally, confirmatory EGD and histopathology results are not captured in the laboratory database, and therefore are not directly available for the patients in this study. EGD referral is managed by the patient’s ordering provider and is outside the scope of laboratory services. As such, the proportion of EG-positive patients who adhered to recommended EGD and those with confirmed BE or EAC on histopathology could not be determined in this real-world cohort. To address this gap, an ongoing registry (ClinicalTrials.gov identifier: NCT05965999) is prospectively tracking EGD and histopathology outcomes, and these findings will be reported in a separate manuscript. The diagnostic performance of the test should be interpreted in the context of prior clinical validation studies [14,15,17,18]. Finally, patient acceptability data were derived from a voluntary, anonymous survey completed by only 586 patients (5.2% of those with successful cell collection). These responses may be subject to self-selection and non-response bias and may not fully represent the experience of the broader tested population.

## 5. Conclusions

In conclusion, this study represents the largest real-world evaluation of non-endoscopic BE testing using EG/EC to date. The EC device was well tolerated and efficient, with a low rate of reported device-related events, supporting its use in both high-volume and routine clinical settings. EG results were generally consistent with the population risk profile. Logistic regression identified age >50 as a strong predictor of test positivity, likely reflecting increased disease prevalence. Overall, these findings support the feasibility of EG/EC for large-scale implementation and highlight their potential to expand access to BE screening in high-risk patients across diverse healthcare settings.

## Figures and Tables

**Table 1:** Baseline Characteristics for Patients with Successful EsoCheck Cell Collection.

Risk Factor	N = 11,355	Percentage (%)
Mean age in years (±SD)	Age: 53.0 ± 14.7 years	
Age > 50 years	6706	59.1%
Male	7815	68.8%
White	8283	72.9%
GERD-associated symptoms	7303	64.3%
Mean body mass index/BMI (±SD; kg/m^2^)	30.4 ± 6.3	
Obese (defined as BMI ≥ 30 kg/m^2^)	5097	44.9%
History of Tobacco Smoking	4989	43.9%
Family History of BE or EAC	546	4.8%
Met at least one of the two U.S. gastroenterology society recommendations for BE screening	9466	83.4%

Gastroesophageal reflux disease (GERD); Body mass index (BMI); BE (Barrett’s esophagus); Esophageal adenocarcinoma (EAC);.

**Table 2:** Device Complaints/Malfunctions Reported in the Quality Management System from Jan 2023 to Jun 2024.

Failure Mode	Quantity	% (n/N) N = 11,991
Hole in Catheter *	1	0.01%
Balloon Burst *	1	0.01%
Balloon Microtear	2	0.02%
Balloon did not fully encapsulate on removal	22	0.18%
Syringe Sticking *	1	0.01%
Missing Stopcock *	1	0.01%
Deformed Stopcock *	1	0.01%

* Device failures identified prior to patient exposure; in these instances, the cell collection was performed with a replacement device. As part of standard operating procedures, Lucid personnel always perform EsoCheck function checks prior to device administration. This allows identification of malfunctions and replacement of most devices before patient exposure.

**Table 3:** Risk Factor Prediction Model for EsoGuard Positivity Utilizing Multivariable Logistic Regression.

Risk Factors	Odds Ratio	95% Lower	95% Upper	*p*-Value
GERD-associated symptoms	1.05	0.94	1.18	0.3706
**Age > 50**	**5.59**	**4.86**	**6.44**	**<0.0001**
Male	1.11	0.99	1.24	0.0689
White	0.97	0.86	1.09	0.5678
**BMI ≥ 30**	**0.82**	**0.74**	**0.91**	**0.0001**
**Smoking history**	**1.33**	**1.20**	**1.48**	**<0.0001**
**Family history of BE/EAC**	**1.42**	**1.14**	**1.78**	**0.0020**

Gastroesophageal reflux disease (GERD); Body mass index (BMI); BE (Barrett’s esophagus); Esophageal adenocarcinoma (EAC); Odds ratio (OR). Area under the curve (AUC)/C-statistic for the model: 0.694; validated AUC: 0.691. Grey-shaded rows indicate risk factors with *p* < 0.05.

## Data Availability

Aggregate data supporting the findings of this study may be made available from the corresponding author upon reasonable request, subject to a data use agreement and applicable privacy regulations.

## Abbreviations

The following abbreviations are used in this manuscript:

BE	Barrett’s Esophagus
SIM	Specialized Intestinal Metaplasia
EAC	Esophageal Adenocarcinoma
EG	EsoGuard
EC	EsoCheck
GERD	Gastroesophageal Reflux Disease
EGD	Esophagogastroduodenoscopy
ACG	American College of Gastroenterology
AGA	American Gastroenterological Association
NGS	Next-Generation Sequencing
*VIM*	Vimentin
*CCNA1*	Cyclin A1
m*VIM*	Methylated Vimentin
m*CCNA1*	Methylated Cyclin A1
PPV	Positive Predictive Value
NPV	Negative Predictive Value
QNS	Quantity Not Sufficient

## Appendix A

**Table A1:** Gag Response Scoring.

**Score**	**Severity of Gag Response**	**Detailed Description**
1	None to minimal	No gagging or patient only gagged during the initial swallow
2	Mild	Non-forceful, infrequent gagging during the procedure (occurring a few times but not through-out the entirety)
3	Moderate	Non-forceful gagging through-out the procedure
4	Severe but able to complete cell collection	Frequent gagging with intense forceful retch, but still able to complete the cell collection
5	Unable to complete cell collection	Unable to complete the cell collection whether due to retching or not

**Table A2:** Results of the Patients Satisfactory Survey.

**Score**	**Total (N = 586 *)**	**Percentage n (%)**
**Pre-Procedure Anxiety Level**		
1–3	278	47.6%
4–6	118	20.2%
7–10	188	32.2%
Not Reported	2	N/A
**Discomfort Experienced During the Cell Collection**		
1–3	482	82.4%
4–6	102	17.4%
7–10	1	0.2%
Not Reported	1	N/A
**Overall Difficulty with the Cell Collection**		
1–3	482	82.3%
4–6	100	17.1%
7–10	4	0.7%
Not Reported	0	N/A
**Willing to recommend EC to others?**		
Yes	560	95.6%
No	26	4.4%
Not Reported	0	N/A
**Willing to undergo EC again if clinically indicated?**		
Yes	510	94.6%
No	29	5.4%
Not Reported	47	N/A

* Not all surveys were completed in their entirety. Missing data was not imputed.
